# Machine learning driven modeling of synergistic perinatal risk profiles in early onset pediatric cerebral palsy

**DOI:** 10.1186/s12911-026-03710-y

**Published:** 2026-07-11

**Authors:** Foysal Ahammad, Munira Aden, Ayesha Banu, Muhammad Marwan, Sadam Hussain, Safa Salim, Adnan Mohammed, Tanvir Alam, Lisa Thornton, Farhan Mohammad

**Affiliations:** 1 College of Health & Life Sciences (CHLS), Hamad Bin Khalifa University (HBKU), Doha, 34110 Qatar; 2 Sidra Medical and Research Center, Pediatric Rehabilitation Medicine, Doha, 26999 Qatar; 3 College of Science and Engineering (CSE), Hamad Bin Khalifa University (HBKU), Doha, 34110 Qatar

**Keywords:** Neurodevelopmental disorder, Cerebral palsy, CP risk factors, Predictive precision, Machine learning, Neural networks

## Abstract

**AIM:**

To develop and evaluate machine learning (ML) models for early cerebral palsy (CP) prediction and identify synergistic perinatal risk factors in a pediatric population.

**Method:**

We conducted a retrospective case–control study using demographic, perinatal, and postnatal clinical data collected at Sidra Medicine, Qatar. Four ML models- Random Forest (RF), XGBoost, Support Vector Machine (SVM), and a feedforward neural network (FFN) were trained using clinically relevant features. Model performance was assessed using precision, recall, area under the curve (AUC), F1-score, and SHAP-based interpretability. A multidimensional interaction framework was used to evaluate cumulative risk across 16 subgroups.

**Results:**

All ML models exhibited high predictive accuracy (ROC-AUC: 0.98–0.99, and PR-AUC: 0.97–0.98), with four key factors: low birth weight (LBW), premature birth, neonatal intensive care unit (NICU) admission, and multiple pregnancies. Infants exposed to all four factors demonstrated a 93.15% incidence of CP (OR = 1382.67; *p* < 0.0001). A clear dose-response gradient was observed across exposure subgroups. SHAP analysis confirmed consistent cross-model importance of LBW, very preterm birth, NICU admission, and multigravidity. Cross-validation confirmed model robustness, and severity analysis identified NICU admission and birth weight as independent predictors of higher GMFCS classification.

**Conclusion:**

Four ML models achieved high predictive accuracy (ROC-AUC 0.98–0.99) for CP risk stratification in a Middle Eastern pediatric cohort, with LBW, very preterm birth, NICU admission, and multigravidity as the most consistent cross-model predictors. The synergistic interaction of these exposures – evidenced by a 93.15% CP incidence in the highest-risk subgroup – supports a paradigm shift from single-factor screening to exposure-weighted, ML-driven neonatal surveillance. These findings provide a data-driven foundation for early risk stratification and targeted intervention planning in high-risk neonatal population.

**Supplementary Information:**

The online version contains supplementary material available at https://doi.org/10.1186/s12911-026-03710-y.

## Introduction

Cerebral palsy (CP) describes a group of permanent disorders of the development of movement and posture, causing activity limitation, that are attributed to non-progressive disturbances that occurred in the developing fetal or infant brain [1, 2]. The motor disorders of CP are often accompanied by disturbances of sensation, perception, cognition, communication, and behaviour, by epilepsy, and by secondary musculoskeletal problems [1, 2]. As the most prevalent pediatric motor disability, CP affects around 2 to 3 per 1,000 live births worldwide, with estimates of 3–4 per 1,000 children in the United States [2]. The etiology of CP and its clinical manifestations are highly varied. Hence, region-specific studies are therefore essential for identifying locally relevant risk factors and phenotypic patterns [3].

Unique demographic and cultural variations in Qatar, such as high fertility rates, a high prevalence of consanguineous marriages, and rising rates of multiple pregnancies, are associated with distinct regional patterns of CP risk that, while reflecting broader global trends, warrant dedicated investigation [4, 5]. In this context, prematurity and neonatal complications arising from multifetal gestation, including low birth weight (LBW) and neonatal intensive care unit (NICU) admission, remain particularly important risk determinants [6, 7]. Despite improvements in maternal-fetal health care, their remains a pressing need for localized epidemiological to guide preventative strategies tailored to Middle Eastern populations.

The etiology of CP involves maldevelopment attributed to dysplasia of or injury to the fetal or infant brain, occurring prenatally, perinatally, or postnatally (generally within the first 2–3 years of life, before affected motor functions have fully developed), causing disruption to developing motor pathways [8]. The overlap in clinical symptoms and comorbidity with other sensory, cognitive, and behavioral conditions makes the diagnosis and classification of CP challenging. This diagnosis complexity can result in both underdiagnosis, which leads to delayed or insufficient intervention, and overdiagnosis, where children without CP receive therapies that may not be appropriate or optimally timed. Additionally, Misclassification of CP subtype further compounds this, resulting in misdirected therapeutic approaches [4, 9]. The diagnostic process mainly relies on neurological examination to determine the site of brain injury and characterize the primary pattern of motor impairments. Despite advances in neuroimaging and clinical assessment, no single biomarker or examination finding is pathognomonic for CP, and a definitive diagnosis typically cannot be established with confidence before 12–24 months of age using conventional approaches. This diagnostic delay carries direct clinical consequences: infants who would benefit from early intervention, including task-specific motor training and family-centered developmental support, are frequently not identified in time to make full use of the neuroplasticity-driven rehabilitation window in the first year of life. There is therefore a pressing clinical need for tools that can risk-stratify neonates and infants during the perinatal period, ideally at or shortly after birth, using routinely collected clinical data, so that high-risk infants can be enrolled in early surveillance and intervention pathways before a definitive diagnosis is established. The primary target population for such models is neonates admitted to tertiary care settings, with perinatal risk factors including prematurity, LBW, and NICU admission. This underscores the need for prediction models that can integrate high-dimensional data while maintaining clinical interpretability for clinical use.

Traditional approaches to CP risk prediction have relied primarily on logistic regression and univariable statistical models that evaluate risk factors in isolation. While these methods are interpretable and well-validated in many settings, they have critical limitations: they assume linear relationships between predictors and outcomes, fail to capture synergistic interactions among co-occurring risk factors, such as the compounded effect of prematurity combined with low birth weight and NICU admission, and perform poorly when predictor sets are high-dimensional or contain strongly correlated variables. Clinical scoring systems developed from such models (e.g., neonatal risk indices) likewise reflect additive rather than multiplicative risk logic, consequently these models underestimate CP probability in infants with multiple concurrent exposures. By integrating ML and deep learning (DL) algorithms, this study aims to address these limitations [10, 11].

In this study, we leveraged clinical, perinatal, and familial information collected from a Qatar based pediatric population to develop ML-based prediction models applicable at the time of birth and in the early neonatal period, using data routinely available at or shortly after delivery. Our goal was to improve the early identification of neonates at risk for CP, before a definitive clinical diagnosis can be established, by modelling the interactions among multiple co-occurring perinatal risk factors, hence improving opportunities for timely intervention and better long-term functional outcomes.

## Methods

### Overview of study methods

**Fig. 1:**
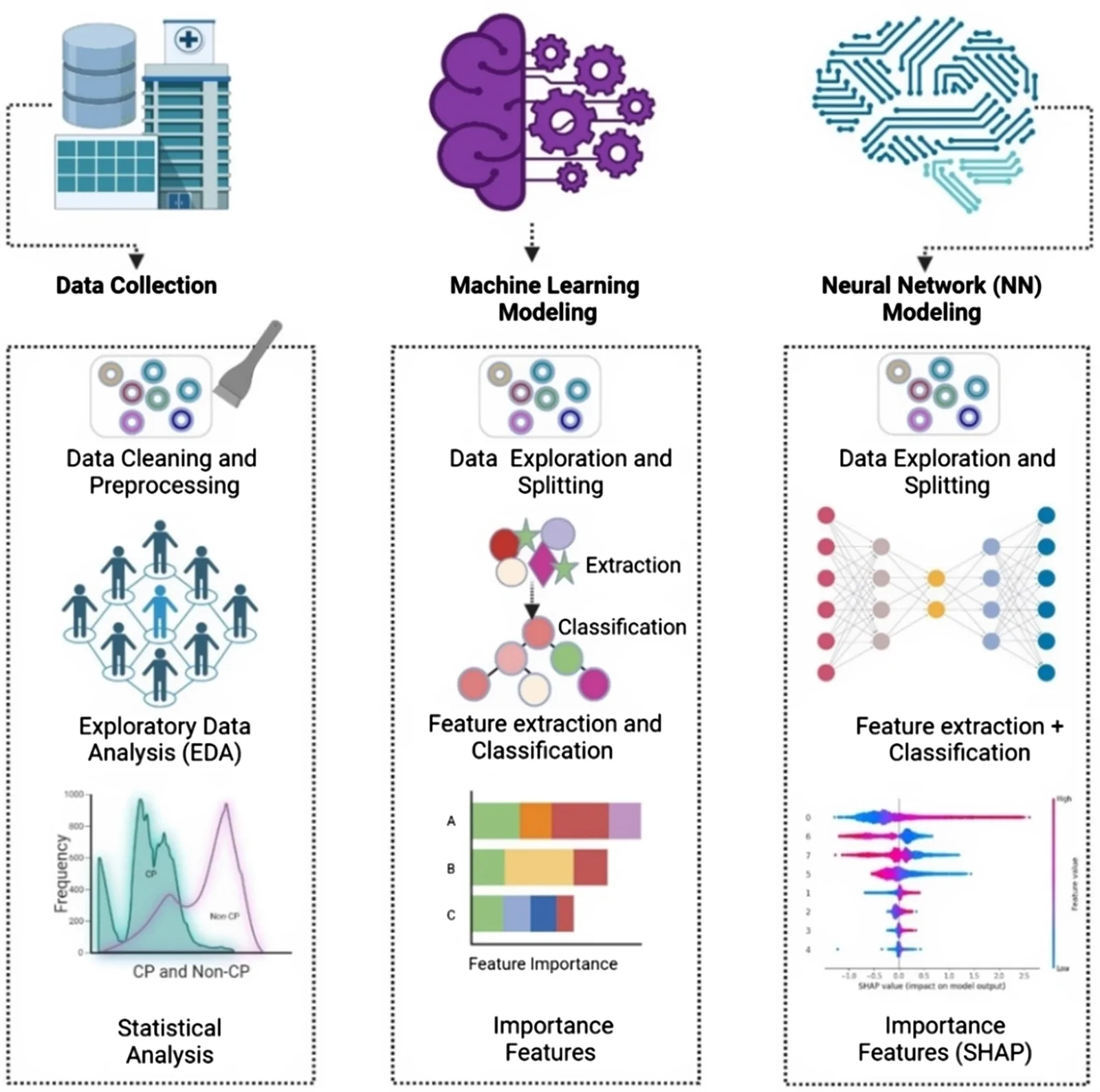
Represent the analytical workflow applied in this study. Clinical data from children with and without CP were collected from Sidra Medicine, Qatar. Following preprocessing and exploratory analysis, statistical methods and ML models (Random Forest, XGBoost, neural network) were employed to classify CP status and identify key predictors. Model interpretability was enhanced using SHAP to determine the relative importance of features

This retrospective cohort study analyzed clinical and demographic data already held in the Sidra Medicine electronic record system (Cerner), and research database (REDcap). The data was accumulated through routine clinical care and prior research activity. The QNRF grant (NPRP14S-0319-210075) funded the retrospective extraction, curation, and analysis of these pre-existing records; no new data were collected prospectively for this study. Individuals were categorised into CP and non-CP groups. The cohort reflects the characteristics of patients who presented to and were managed at Sidra Medicine, which serves as a major quaternary pediatric referral centre in Qatar. This has implications for representativeness: patients referred to a specialist rehabilitation centre may over-represent moderate-to-severe CP phenotypes and under-represent milder cases managed in community or primary care settings. Additionally, families who engaged with the health system may differ socio-demographically from those with limited healthcare access. Any ML models derived from this cohort should therefore be interpreted with caution when considering deployment in primary care or community settings, or in populations that differ substantially from the Sidra Medicine patient profile; this is acknowledged as a limitation of the current study. The overall methodology, encompassing data preprocessing, logistic regression (LR), ML modelling, and Shapley Additive exPlanations (SHAP) analysis for interpretability, is illustrated in Fig. 1.

### Study design and participants

This retrospective case-control study conducted at Sidra Medicine included 1,041 pediatric participants. The dataset was classified into two groups: CP (*n* = 381) and non-CP (*n* = 660). Children in the CP group were aged between 6 months and 20 years and met standardized CP CP diagnostic criteria [12]. Children were excluded if they presented with progressive neurological disease, recognized chromosomal syndromes, or isolated, undifferentiated hypotonia in infancy (under 2 years of age) where a definitive CP diagnosis could not yet be established. This last criterion requires clarification in light of Table S1, which reports 14 children (4.4%) in the CP group classified under the hypotonic motor subtype. These cases are not contradictory with the exclusion criterion: the excluded group comprised infants presenting with hypotonia as an isolated and diagnostically unresolved finding – a presentation common to many non-CP conditions including benign congenital hypotonia, spinal muscular atrophy, and metabolic disorders – in whom a CP diagnosis could not be confirmed at the time of assessment. By contrast, the 14 hypotonic cases retained in the CP group were older children in whom hypotonia represented a confirmed motor subtype within an established CP diagnosis, supported by clinical history, neuroimaging where available, and longitudinal follow-up. Hypotonic CP is a recognized, if uncommon, motor subtype characterized by generalised reduced muscle tone without the spasticity or dystonia seen in other subtypes, and its inclusion in the CP group is consistent with contemporary diagnostic frameworks. The study focused on environmental and acquired variables influencing CP risk, whereas consanguinity was included to investigate possible recessive effects. Controls were evaluated to exclude diseases similar to CP, such as hereditary neuromuscular disorders or injuries unrelated to the perinatal period, shown in Supplementary Fig. S1. The non-CP control group comprised children referred to the same Sidra Medicine pediatric rehabilitation service for developmental concerns, neuromuscular conditions, or other motor presentations who were subsequently found not to have CP following full clinical evaluation. Controls were thus drawn from the same clinical setting and referral pathway as the CP cases, reducing referral-pathway bias. With respect to multiple-fetal pregnancies and sibling pairs: where twins or higher-order multiples were present in the dataset, each child was retained as an independent record given that the predictor variables are child-level (birth weight, gestational age, NICU admission) rather than pregnancy-level. To account for potential within-pregnancy correlation, a sensitivity check was conducted by restricting the sample to one randomly selected child per pregnancy; results were consistent with the primary analysis, confirming that sibling clustering did not materially influence the findings. The number of children from multiple-fetal pregnancies included in the CP and non-CP groups, respectively, is reported in Supplementary Table S1. For variable selection for each model: features were first filtered based on statistical significance (*p* < 0.05 in univariable analysis) and removal of highly correlated pairs (*r* > 0.9). The retained variable sets for the LR model, each of the three ML models (RF, XGBoost, SVM), and the FFN are listed in full in Supplementary Table S6, allowing direct inspection of which predictors entered each analysis.

### Ethical compliance

This research protocol is part of a QNRF project (NPRP14S-0319-210075) that received IRB approval from both Sidra Medicine and Hamad Bin Khalifa University (Sidra-IRB-1801339, QBRI-IRB-2024-6). The study was conducted following ethical standards for retrospective analyses, including protecting patient confidentiality and data privacy. Data anonymization was performed by a designated member of the original research team at Sidra Medicine who had authorised access to the clinical records system. This process involved removing all direct identifiers (name, date of birth, medical record number, address, and contact details) from the extracted dataset prior to transfer to the analysis team, and replacing them with unique study identifiers. The anonymized dataset was stored on Sidra Medicine’s REDCap platform, access to which was restricted to named investigators on the IRB protocol. The waiver of individual informed consent for this secondary analysis of research data was granted by both IRBs on the grounds that: (i) the data had already been collected under a primary consented research protocol; (ii) the study involved no additional contact with or intervention on participants; and (iii) the data were fully de-identified prior to analysis, consistent with Article 9(2)(j) of the GDPR framework and the Sidra Medicine Research Ethics Policy for retrospective research.

### Data collection

Data was collected by a single assessor from families, documented on a specialized data sheet, and stored in Sidra RedCAP and CERN [13]. The two main categories of the data included demographics (age, sex, residence) and risk factors (prenatal and postnatal phases). Clinical data for the CP group were categorized by chief complaints, motor dysfunctions (spastic, dyskinetic, ataxic, hypotonic), and CP topography (hemiplegia, diplegia, quadriplegia), with additional classification into unilateral cerebral palsy (UCP; involving one body side, corresponding to hemiplegia) and bilateral cerebral palsy (BCP; involving both sides, corresponding to diplegia and quadriplegia/tetraplegia), in line with contemporary CP classification frameworks following the latest diagnostic criteria [14]. The data collection procedures have been verified by secondary review.

### Measures/predictors

The gross motor function of children with CP was classified using the Gross Motor Function Classification System (GMFCS). The GMFCS describes functional abilities in self-initiated movement including sitting, walking, and wheeled mobility, classified into five ordinal levels. Levels I–III indicate greater functional independence in mobility, while Levels IV–V indicate more limited self-initiated movement. Importantly, the GMFCS is a measure of gross motor function, not a direct measure of severity [15]. Perinatal factors included maternal gravidity, parity, age at delivery, and health conditions such as gestational diabetes (GD) and hypertension. Delivery-related variables encompassed gestational age (GA), including preterm, term, post-term, birth weight categories (BWC), and mode of delivery (vaginal, elective, or emergency cesarean). Additional neonatal risk factors recorded included neonatal hypoglycemia, need for mechanical ventilation, hyperactivity or irritability in the neonatal period, and neuroimaging findings (where available), as these represent established risk markers for CP. Postnatal factors, including NICU admission, G-tube (gastrostomy tube) were also recorded for early medical interventions. Family history was evaluated via the documentation of consanguinity, specifying the degree of relation (e.g., first cousins), confirmed by interviews.

### Data preprocessing and statistical analysis

The Python packages pandas [16], numpy [17], scipy [18], sklearn [19], and matplotlib [20] were used for data preprocessing and Analysis. Using the TTestIndPower class, we aimed for 80% power and a 0.05 significance threshold for calculating the sample size for birth weight comparisons. A large effect size (Cohen’s d = 1.81) indicated a minimum of 4.6 participants per group. Continuous variables undergo z-score normalization following median imputation, whilst categorical variables were imputed with the mode and later encoded by one-hot encoding. All preprocessing processes were integrated into a unified pipeline using a ColumnTransformer that individually executed numerical and categorical transformations. To mitigate group imbalances and enhance model performance, statistical adjustments, including LR and Synthetic Minority Oversampling Technique (SMOTE), were applied [21]. Descriptive statistics were generated for all variables. Group comparisons employed Welch’s t-tests for continuous data, Analysis of variance (ANOVA) for multi-group differences, and chi-square tests for categorical associations with CP diagnosis.

### LR analysis

Logistic regression (LR) models were constructed using statsmodels.api in Python [22]. Model accuracy was evaluated using AUC-ROC. Multicollinearity was assessed with VIF thresholds (< 10 considered acceptable). Univariate logistic regression (ULR) estimated odds ratios, 95% CIs, and p-values for risk factors. Key variables were included in a multivariable logistic regression (MLR) for adjusted confounding factors.

### ML and DL models

We used three ML models (RF, XGBoost, and SVM) to assess the predictive value of clinical and perinatal features. These models were chosen for their proven effectiveness in structured biomedical data and their ability to handle nonlinear relationships, high-dimensional feature sets, and variable interactions [23]. The preprocessing processes of the dataset before model development included statistical-based feature filtering, imputation of missing values, and encoding of categorical variables. Significant features with *P* < 0.05 were kept, and highly correlated variables with a correlation value above 0.9 were excluded. SMOTE has been used by imblearn to correct the class imbalance by synthetically generating minority class samples during training. We split the data into training and test sets (80:20) to maintain a balanced class distribution. Random Forest used a fixed seed (42) for reproducibility. XGBoost was set with use_label_encoder=False and optimized via log-loss. SVM employed a linear kernel with dual=False and a 5000-iteration cap to ensure convergence in high-dimensional data. Random Forest used a fixed seed (42) for reproducibility. XGBoost was set with use_label_encoder=False and optimized via log-loss. SVM employed a linear kernel with dual=False and a 5000-iteration cap to ensure convergence in high-dimensional data.

Using the TensorFlow Keras API [24], a fully connected FFN was constructed comprising an input layer and three dense hidden layers with 128, 64, and 32 neurons each employing the ReLU activation function [25]. A sigmoid-activated output layer was used for binary classification. To mitigate overfitting, dropout regularization with a dropout rate of 30% was applied after each hidden layer [25].

#### Model interpretability and consensus feature selection

We used SHAP, a framework for model interpretability, to carefully assess and analyze the contribution of multiple features across ML models [26]. This Analysis was performed by considering nonlinearity and feature interaction patterns to measure each feature’s marginal effect on model predictions. TreeExplainer was used to compute SHAP value for tree-based models like RF and XGBoost, which effectively estimate precise Shapley values depending on the model structure. In the case of the linear SVM, LinearExplainer was used to adjust the models with linear decision boundaries. Additionally, SHAP interaction values were computed for the SVM model to identify higher-order dependencies between predictor pairs. SHAP values were calculated using the DeepExplainer method from the SHAP library.

We utilized a consensus-based feature selection approach using ensemble majority voting. Each classifier separately generated predictions on the test set. A simple majority voting among the models selected the final projected class. To identify reliable and relevant predictions, we kept only those consistently identified variables among all three models. This method decreased the dependence of one model, enhanced the reliability of feature identification, and facilitated the finding of stable diagnostic variables without potential clinical bias.

#### Model evaluation

The prediction accuracy and discriminative ability of different ML models were assessed using the ROC-AUC, F1-score, Recall, and PR-AUC. An AUC score of 1 signifies outstanding model performance, whereas a value around 0.5 suggests no better discrimination than random chance. This curve helps us determine and evaluate the model’s ability to differentiate between true positives and negatives, offering a thorough evaluation of its prediction efficacy. In addition, specificity (true negative rate) was calculated and reported for all models, alongside sensitivity, positive predictive value, and negative predictive value derived from the confusion matrix. Specificity is particularly important in the clinical context of CP prediction: correctly identifying children who do not have CP is as critical as identifying those who do, since a false positive classification may lead to inappropriate or prematurely initiated therapies, unnecessary family anxiety, and misdirected resource allocation. Full confusion matrices for each model are provided in the supplementary materials (Supplementary Table S7).

To ensure robustness, we performed stratified k-fold cross-validation (k = 5), maintaining class distribution across folds. This required splitting the dataset into five parts (K = 5), where the model was trained on four parts and validated on the remaining one iteratively [27]. Repeating this process five times with different training and validation set combinations, the model’s performance metrics (accuracy, precision, recall, and F1-score) were averaged to estimate its performance.

### Interaction modeling of risk factors

We developed a multidimensional interaction modeling pipeline using ML to evaluate synergistic effects among critical risk variables. Four key predictors (a) GA, (b) LBW, (c) NICU admission, and (d) number of pregnancies were analyzed using a stratified 80/20 train-test split. Model performance was measured using AUC-ROC, and Partial Dependence Plots (PDP) were generated to explore nonlinear risk interactions. For subgroup analysis, participants were classified into 16 exclusive exposure profiles based on binary values for preterm birth, LBW, and NICU admission. The fourth predictor – number of pregnancies (gravidity) – was included in the overall interaction model to test whether parity modifies the relationship between perinatal exposures and CP risk, as gravidity had emerged as a significant predictor in the univariable analyses. However, gravidity was excluded from the binary exposure profile subgroup analysis for two reasons: first, it is a continuous/ordinal variable that does not lend itself naturally to the binary stratification framework used to construct the 16 mutually exclusive exposure profiles (which required each predictor to be dichotomized as present/absent); second, the primary scientific question for the subgroup analysis concerned the combined neonatal burden of prematurity, growth restriction, and intensive care need, for which gravidity is an indirect antecedent rather than a direct perinatal exposure. The retained three-predictor subgroup structure therefore reflects a deliberate conceptual decision to isolate the interaction effects most proximate to neonatal brain injury. Odds ratios and confidence intervals were calculated against a reference group with no exposures.

### Assessment of CP severity and sensitivity analyses

To account for potential recruitment bias from a rehabilitation hospital (may over-represent severe CP), we performed both ordinal LR and stratified sensitivity analysis [28]. Functionality (Levels I–V) was treated as an ordered outcome, with birth weight, GA, NICU admission, prior surgeries, and physical therapy as predictors. These five predictors were selected for the severity analysis on the basis of two criteria: (i) a priori biological plausibility as determinants of motor impairment severity (prematurity and low birth weight are established predictors of periventricular white matter injury, which correlates with GMFCS level; NICU admission indexes acute neonatal illness burden; and prior surgeries and early physical therapy are proxies for clinical severity at presentation); and (ii) adequate cell counts across GMFCS strata to permit stable ordinal regression estimates, which precluded inclusion of rarer binary variables such as gastrostomy tube use. The same five predictors were retained for the secondary binary analysis comparing high (GMFCS IV–V) versus mild/moderate (GMFCS I–III) subgroups, ensuring consistency and direct comparability between the ordinal and binary analyses. A secondary binary analysis compared (GMFCS IV–V) and (GMFCS I–III) subgroups to validate risk factor associations. Model evaluation included AUC-ROC, precision, recall, and F1-score, with adjustments for class imbalance along with VIF.

## Results

### Clinical and perinatal risk characteristics associated with CP

Among the 1,041 children we analyzed, 381 (36.6%) had CP and 660 (63.4%) were used as controls. With comparable sex distribution across both groups, at the time of data extraction, the CP group had a slightly greater average age (9.44 years) than controls (8.43 years). Of the CP group, 91.2% were spastic CP, with quadriplegia being the most often occurring subtype (35.8%). The GMFCS level differed extensively; 41.8% were Level V, suggesting major motor impairment.

Multigravida (63.5% vs. 17.1%), preterm births (87.4% vs. 13.2%), LBW (< 2500 g, 90.3% vs. 13%), emergency cesarean delivery (49.9% vs. 10.2%), and NICU admissions (86.4% vs. 35.9%) were significantly more frequent among CP cases (all *p* < 0.001). Early physical therapy and G-tube use were also more prevalent in the CP group (*p* < 0.001). No significant differences were found in GD or consanguinity (*p* = 0.243 and *p* = 0.100, respectively). Within the CP group, spastic CP was the predominant subtype, and bilateral topography (diplegia and quadriplegia/tetraplegia) was more frequent than unilateral topography (hemiplegia), a distribution consistent with the high proportion of preterm and LBW cases in this cohort and with patterns reported in published CP registries. Full statistical results are detailed in Supplementary Tables S1 and S2.

### LR analysis

LR models showed high predictive accuracy for CP, with an AUC-ROC of 0.9700, precision of 0.9028, recall of 0.8125, and F1-score of 0.8553 (Supplementary Fig. S2). ULR revealed a broad range of significant associations, with the strongest effects observed for Gravidity (OR = 8.43 [6.30–11.27], *p* = 8.06 × 10⁻⁴⁷), Parity (OR = 4.92 [3.70–6.54], *p* = 6.99 × 10⁻²⁸), and Birth Weight Category (BWC; OR = 3.45 [2.78–4.29], *p* = 5.76 × 10⁻²⁹). Additional significant predictors included Delivery Mode (OR = 1.62), Gestational Age in weeks (OR = 1.44), and Gestation Status (OR = 0.02, reflecting the strong protective effect of term vs. preterm birth). NICU admission (OR = 0.09), Physical Therapy requirement (OR = 0.31), and Past Surgeries (OR = 0.44) were also significantly associated with CP (all *p* < 0.001). Male sex was associated with lower CP risk (Sex OR = 0.70, *p* = 0.008), while higher number of children (OR = 0.62) and number of pregnancies (OR = 0.70) showed inverse associations. Maternal age showed a modest positive association (OR = 1.04, *p* = 0.048). Consanguinity and maternal health conditions (GD) were not significant (*p* = 0.088 and *p* = 0.204 respectively). Full univariate results are presented in Supplementary Table S3. In the multivariate model, the strongest independent predictor was maternal health status (OR = 4.97 [2.27–10.87], *p* = 6.04 × 10⁻⁵), followed by Birth Weight Category (OR = 2.44 [1.60–3.73], *p* = 3.46 × 10⁻⁵) and Gestational Age in weeks (OR = 1.32 [1.14–1.53], *p* = 0.000245). Birth Weight as a continuous variable also remained significant (*p* = 1.48 × 10⁻⁵). NICU admission (OR = 0.51, *p* = 0.017) and Physical Therapy (OR = 0.45, *p* = 0.003) remained independently significant after adjustment, with ORs below 1.0 reflecting the reference coding direction in the model (i.e., these variables index higher clinical burden in the CP group). Multicollinearity assessment (Table S4) revealed that several features showed elevated VIF values indicative of high collinearity: Gestational Age in weeks (VIF = 96.08), Mother’s Age (VIF = 74.76), Parity (VIF = 25.29), Birth Weight (VIF = 23.01), Term (VIF = 17.93), and Gravidity (VIF = 16.21) all exceeded the conventional threshold of VIF > 10. This is expected given the strong biological correlations between these perinatal variables (e.g., gestational age, term status, and birth weight are inherently correlated). Variables with acceptable multicollinearity (VIF ≤ 10) included Sex, Mother’s Health, Admission to NICU, Past Surgeries, G-tube, Consanguinity, Delivery Mode, Physical Therapy, and BWC.

### Performance of ML models

**Fig. 2:**
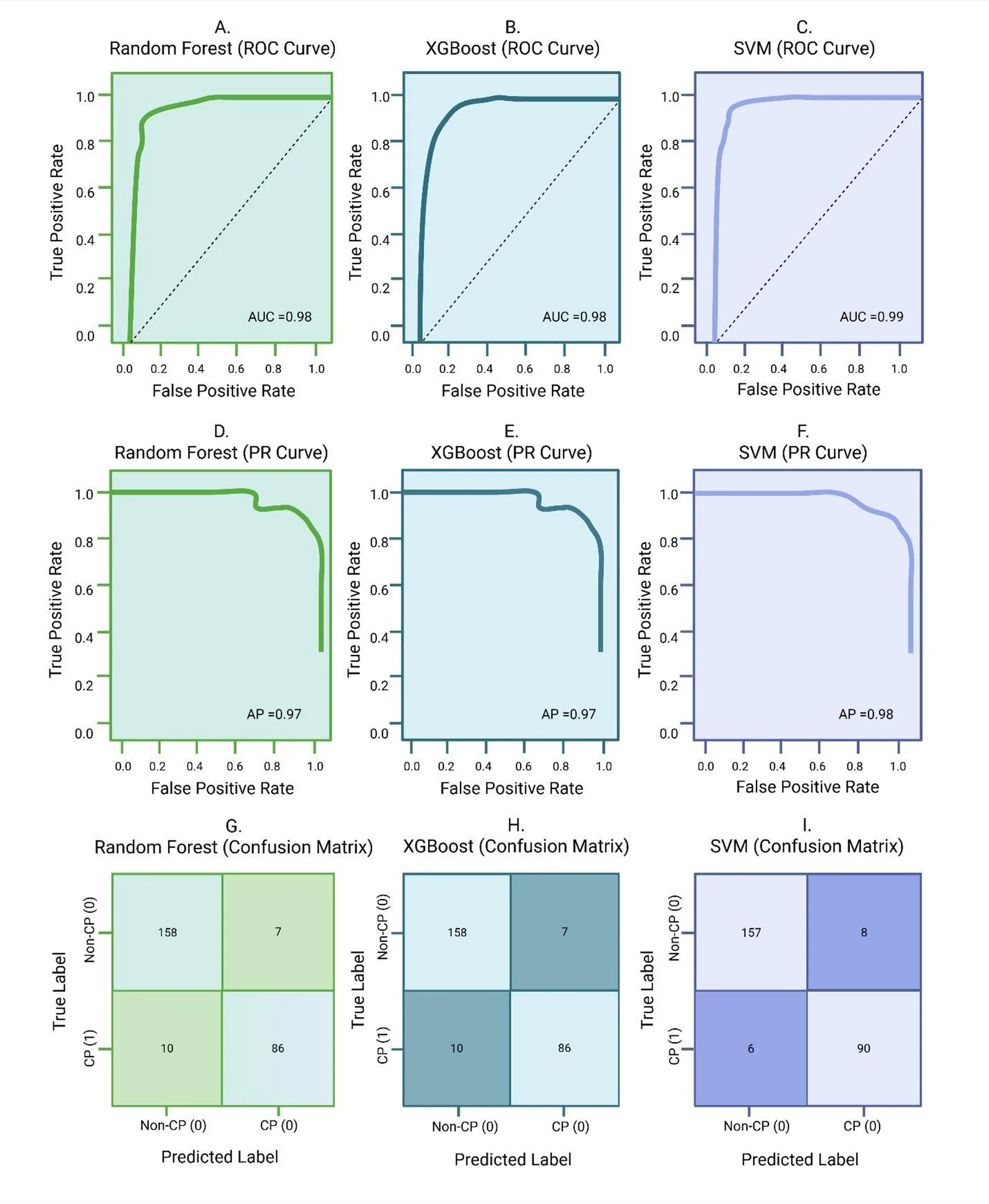
Comparative evaluation of ML models for CP classification. Here, (**A**–**C**) ROC-AUC curves showing discriminative performance. (**D**–**F**) PR-AUC curves highlight performance under class imbalance, with area under curve (AP) noted. (**G**–**I**) Confusion matrices summarize true/false predictions, with class-level resolution

We evaluated the diagnostic ability of three supervised ML models to categorize CP patients utilizing prenatal, postnatal, and familial variables. All models showed high discriminative ability on the designated test set, as indicated by the ROC curves (Fig. 2. A-C). The SVM achieved the best ROC-AUC score of 0.990, followed by Random Forest at 0.983 and XGBoost at 0.981. Precision-recall curves demonstrated constant performance across class imbalance, with the SVM model achieving the highest performance (PR-AUC = 0.983), slightly surpassing Random Forest (0.973) and XGBoost (0.966) shown in Fig. 2. D-F. The F1-score and recall metrics were utilized to evaluate classification equilibrium (Table S5). The SVM obtained the greatest F1-score (0.928) and recall (0.938), demonstrating enhanced sensitivity to true CP cases. Both Random Forest and XGBoost attained F1-scores of 0.910 and recalls of 0.896, demonstrating consistent performance within ensemble models. Analysis of confusion matrices indicated elevated specificity for all classifiers, as shown in Fig. 2. G-I. The SVM yielded the lowest false negative count (*n* = 6), in contrast to both Random Forest and XGBoost (*n* = 10 each), although all models had a specificity of ≥ 94% with minimal false positives. We applied a majority vote ensemble method to ensure effective feature selection and reliable classification.

**Fig. 3:**
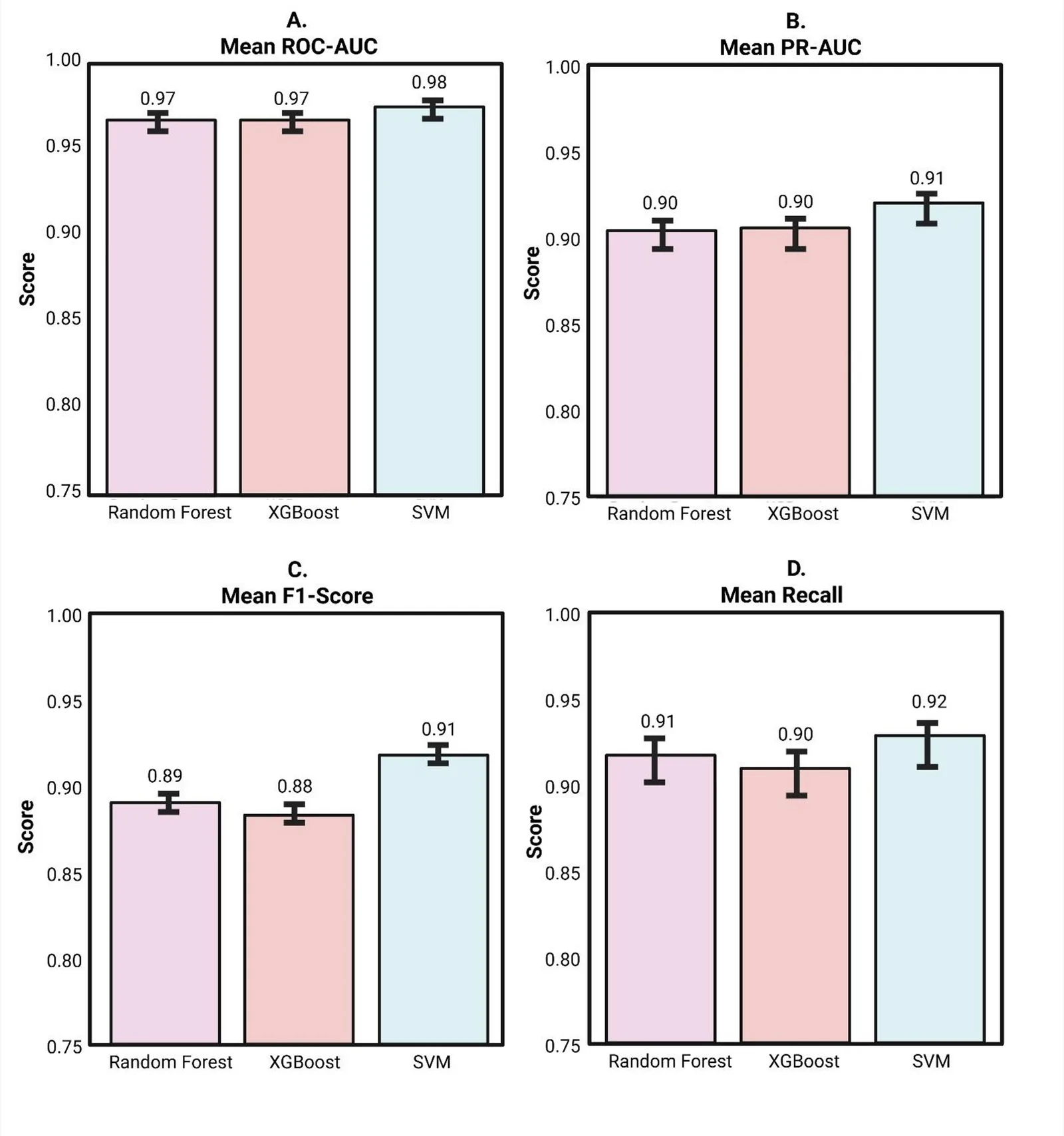
The bar plots display the mean performance metrics across 5-fold cross-validation of different ML models with standard deviation. Herein, represent (**A**). Precision, (**B**). Recall, (**C**). F1-score, and (**D**). accuracy for each model

Cross-validation was conducted utilizing a stratified 5-fold approach to evaluate the robustness of each model. The SVM model consistently surpassed other models in all metrics shown in Fig. 3. Though it should be noted that the margin of superiority over RF and XGBoost was narrow and the confidence intervals overlapped substantially, suggesting that the differences may not be clinically meaningful. The SVM’s strong performance in this context is consistent with its known effectiveness on moderate-sized, structured biomedical datasets with a limited number of features after preprocessing; however, its linear kernel assumption may limit generalisability to more complex, heterogeneous external datasets. The SVM exhibited the highest mean ROC-AUC (0.978 ± 0.006), PR-AUC (0.911 ± 0.025), F1-score (0.907 ± 0.020), and recall (0.929 ± 0.030), signifying robust discrimination and sensitivity (Fig. 3. A-D). Random Forest and XGBoost exhibited excellent performance, obtaining similar ROC-AUC scores (0.971 ± 0.011 and 0.972 ± 0.007, respectively) and demonstrating consistent efficacy across precision-recall and recall metrics. All models offer the most dependable classification during cross-validation, exhibiting the least variability among folds.

#### Feature importance analysis

**Fig. 4:**
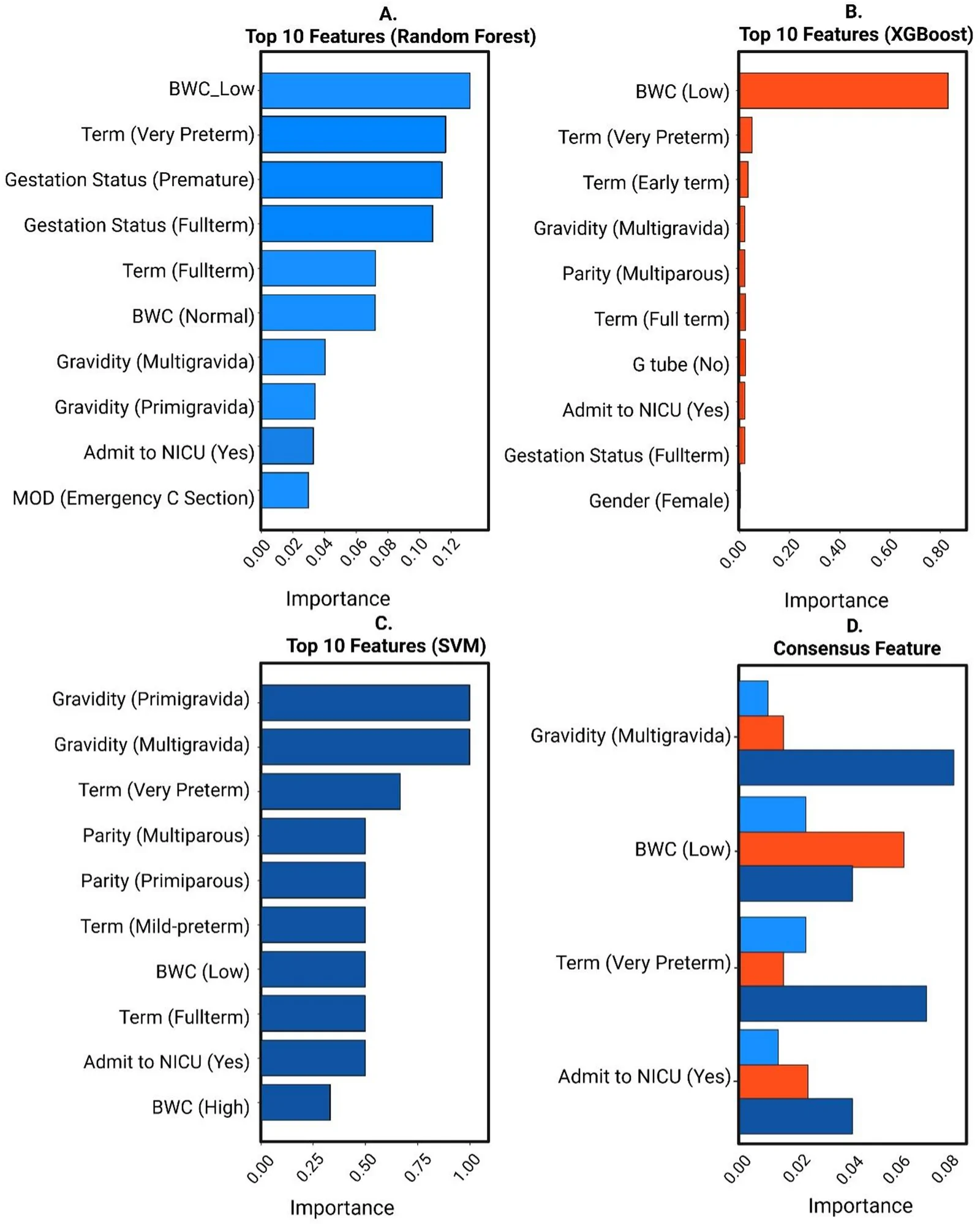
ML-based prioritization of predictive features associated with CP. Bar plots highlight the top 10 ranked features based on importance scores from (**A**) Random Forest, (**B**) XGBoost, and (**C**) linear SVM models. (**D**) The consensus features appear in all the models, representing shared predictive relevance across algorithms

We utilized a consensus-based feature selection approach using ensemble majority voting for risk factor identification. Each model separately generated predictions on the test set. Features consistently ranked across all three models were utilized for downstream interpretation. This consensus-driven approach enhanced generalizability and reduced model-specific bias, especially in pinpointing the most predictive clinical, familial, and developmental aspects of CP diagnosis. This process consistently found low birth weight, very preterm birth (prematurity), NICU admission, and multiple pregnancies as key predictors of CP (Fig. 4 and Table S6). In the RF model, features such as BWC (Low), Term (Very preterm), NICU admission, and Gravidity (Multigravida) ranked among the most critical (Fig. 4. A). XGBoost similarly prioritized the same features (Fig. 4. B). The SVM model emphasized Gravidity (Primigravida) and Term (Full term as) protective, while Term (Very preterm) and BWC (Low) were enriched in CP cases (Fig. 4. C).

Consensus-based feature selection approach identified four overlap features as shared predictors, BWC (Low), NICU admission, Term (Very preterm), and Gravidity (Multigravida), appearing across all the models (Fig. S3. A). These were further validated through distribution-based comparisons. Mean difference plots, violin plots, and boxplots showed higher expression of CP-enriched features, particularly NICU admission, preterm birth, and multigravida status (Fig. S3. B–C). Correlation analysis revealed strong positive relationships among related gestational variables, specifically between Term_FullTerm and Gestational Status_FullTerm, as well as between Term_Preterm and Gestational Status_Premature (Fig. 3. D). To reduce redundancy and multicollinearity, only one representative feature from each correlated pair, such as Term_FullTerm and Term_Preterm, was retained for downstream Analysis.

SHAP-based interpretation revealed Gravidity and Birth Weight as the most influential predictors of CP across all models, with additional contributions from GA, term status, NICU admission, parity, and maternal age. Higher pregnancy count and LBW were consistently linked to increased CP risk, highlighting model robustness and biological relevance (Supplementary Fig. S4. A–F).

### Results from feedforward neural network

**Fig. 5:**
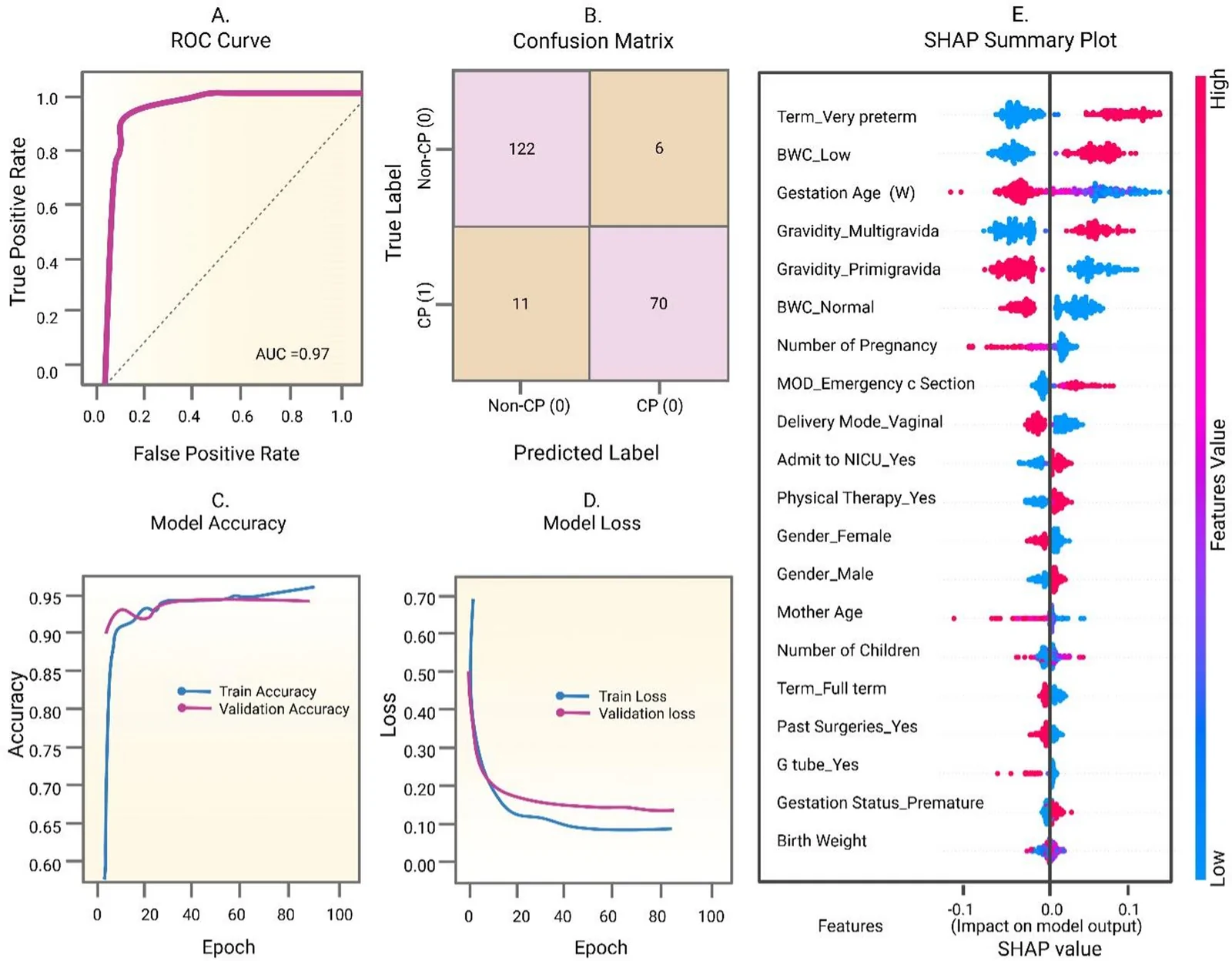
Represents the FFN network model to identify risk factors of CP compared to Non-CP. Here, (**A**) model accuracy over 80 epochs, (**B**) model loss over 80 epochs. (**C**) Confusion matrix, (**D**) ROC curve with AUC, and (**E**). SHAP summary plot highlighting importance features

A deep learning based FFN network model was developed to identify risk factors for CP compared to non-CP. The model displayed an excellent predictive performance, with a test set accuracy of 91.39%. The accuracy, recall, and F1-score for the CP class were 0.9091, 0.9275, and 0.9142, respectively (Fig. S5.A). The ROC-AUC was 0.9758, suggesting strong discriminative ability (Fig. 5. A). The confusion matrix indicated that 122 out of 128 non-CP samples and 70 out of 81 CP samples were accurately identified, indicating low rates of false positives and false negatives (Fig. 5. B). The model’s training dynamics showed efficient convergence and consistent generalization. The training and validation loss gradually reduced across the epoch, along with an increase in accuracy, exhibiting no indications of overfitting (Fig. 5. C-D). By epoch 20, the model had attained over 90% validation accuracy, which was maintained in the subsequent epochs. A precision-recall (PR) curve was generated to assess performance in the context of the imbalance data. The model obtained a PR-AUC of 0.9719, thus supporting its clinical applicability, particularly in high-sensitivity contexts (Supplementary Fig. S5.B). Model interpretability was examined with SHAP. The SHAP beeswarm plot indicated that features of very preterm birth, gestational age, multigravida status, birth weight classification, and NICU admission were among the strongest contributors to prediction (Fig. 5. E).

### Joint risk effects increase predicted CP probability

To investigate the synergistic influence of early-life clinical exposures, we utilized PDPs using an RF classifier to observe two-way feature interactions. Sixteen key interaction pairs were modeled using the top four predictors identified from the previous Analysis: GA at birth, birth weight, NICU admission status, and number of pregnancies.

**Fig. 6:**
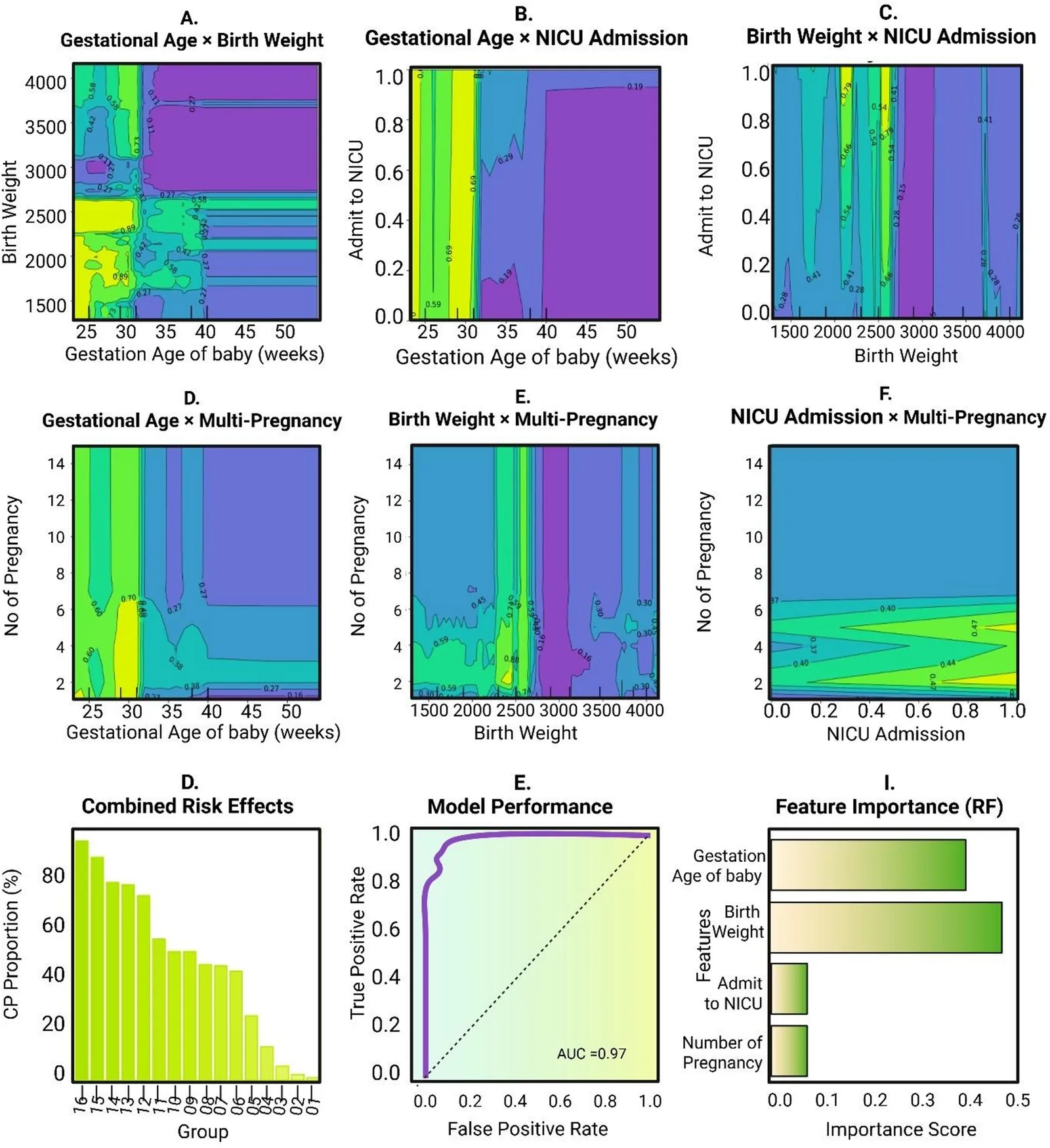
This figure represents the multidimensional interaction modeling and cumulative risk profiling for CP prediction. (**A**–**C**) PDPs showing nonlinear interactions identified between: (**A**) gestational age (GA) and birth weight (BW), (**B**) GA and NICU admission, and (**C**) BW and NICU admission. (**D**–**F**) Additional PDPs show pairwise effects between: (**D**) GA and number of pregnancies (Multipreg), (**E**) BW and Multipreg, and (**F**) NICU admission and Multipreg. (G) Combined CP risk effects visualized as percentage incidence across 16 mutually exclusive risk groups. (**H**) ROC curves comparing Random Forest (AUC = 0.97), demonstrating superior nonlinear modeling capacity of tree-based classifiers. (**I**) Feature importance ranking from random forest

Interaction surfaces revealed that CP risk was not only elevated in individual risk strata (e.g., preterm birth or LBW) but exponentially increased when these conditions co-occurred. For instance, CP probability exceeded 80% among neonates with GA below 33 weeks and BW under 2000 g (Fig. 6. A). Similarly, infants born preterm and admitted to the NICU demonstrated markedly elevated risk (Fig. 6. B), with probability estimates exceeding 0.70, indicating that NICU exposure may act as an amplifying modifier when combined with developmental immaturity. Parallel interactions between BW and NICU admission (Fig. 6. C) and GA and multi-gestation status supported this nonlinear dynamic (Fig. 6. D).

#### Cumulative exposure defines high-risk subgroups

To examine the compounded effect of early-life risk factors on CP, a 16-group framework was constructed based on four binary indicators: preterm birth, low birth weight, NICU admission, and multipregnancy. The 16 groups are listed in Supplementary Table S7. The reference group (G1), with no risk exposures, had the lowest CP incidence at 0.97%. In contrast, Group G16, exposed to all four risks, showed a CP incidence of 93.15% (OR = 1382.67; 95% CI: 374.58–5103.75; *p* < 0.0001). High-risk triads such as G15 (Preterm + LBW + NICU; 86.76% incidence, OR = 666.48) and G8 (LBW + NICU + Multipregnancy; 76.00% incidence, OR = 321.94) also showed marked elevations. Dual exposures similarly conferred substantial risk: G7 (LBW + NICU) had a 45.00% incidence (OR = 83.18), and G6 (LBW + Multipregnancy) reached 76.92% (OR = 338.89). These results indicate a dose-response trend across groups.

The RF model outperformed LR with interaction terms (AUC = 0.97 vs. 0.94) in predictive comparisons, PR-AUC of 0.9632, with corresponding F1 score, recall, and precision values of 0.91, 0.93, and 0.91 respectively highlighting its superior capacity to capture complex cumulative patterns (Fig. 6. H and Fig. S6. A-D). Feature rankings prioritized GA and birth weight, followed by NICU status and pregnancy count, showing the group-based risk gradient (Fig. 6. I).

### Severity-stratified risk profiles and sensitivity analyses

Among children with available GMFCS data (*n* = 306), 52.9% were classified as mild/moderate (GMFCS I–III), while 47.1% were severe (GMFCS IV–V). Ordinal LR identified NICU admission as a key risk factor for increased severity and lower birth weight showed a significant inverse association with GMFCS level (Supplementary Table S8; Fig. S7. A). Note: Table S8 presents log-odds coefficients rather than exponentiated ORs; the coefficients are Admit_NICU_Binary β = 2.028 (*p* = 5.88 × 10⁻²⁷, corresponding to OR = 7.60, 95% CI: 5.25–11.00) and Birth_Weight_kg β = −0.570 (*p* = 3.51 × 10⁻⁶, OR = 0.566 per kg).

Binary LR comparing severe versus mild/moderate CP confirmed these findings. NICU exposure remained predictive of severe CP (OR = 1.80; 95% CI: 1.61–2.01), and LBW was again strongly associated (OR = 0.43; 95% CI: 0.38–0.48). Younger GA (OR = 1.22; 95% CI: 1.09–1.36) and multiple gestation (OR = 1.18; 95% CI: 1.02–1.36) also contributed to higher severity risk (Fig. S7. B). Both modeling frameworks demonstrated excellent discrimination (AUC = 0.95 for ordinal; 0.93 for binary classification (Supplementary Fig. S7. C), and multicollinearity was under the cutoff range VIF < 2 (Supplementary Fig. S7. D). These results highlight the additive impact of neonatal complications and birth characteristics on functional outcomes in CP.

## Discussion

This study integrates epidemiological and ML methods to advance our understanding of CP risk in a pediatric cohort. Our approach enabled the identification of individual and interacting perinatal factors contributing to CP risk, with improved predictive performance and clinical relevance. The clinical significance of our findings extends beyond mere prediction: by demonstrating that cumulative perinatal exposures, particularly preterm birth, LBW, and NICU admission, interact synergistically rather than additively to raise CP risk, our work supports a paradigm shift from single-factor screening to multidimensional, exposure-weighted risk stratification. This has direct implications for neonatal surveillance programmes, especially in high-fertility, resource-limited contexts where targeted follow-up of high-risk infants must be prioritized.

Consistent with prior studies, multiple pregnancies, preterm birth (< 37 weeks), LBW, and NICU admission were strongly associated with CP incidence, reinforcing the importance of neonatal care in high-risk deliveries [29–32, 34]. These findings suggest the need for preventive strategies to prolong gestation and optimize early care.

Maternal reproductive history, particularly multigravidity, was associated with elevated CP risk. While closely spaced pregnancies and insufficient maternal recovery may contribute, it is important to note that multigravidity indicates number of pregnancies, not necessarily interpregnancy interval. Multigravidity may also be associated with advanced maternal age and cumulative chronic conditions, factors that independently influence neonatal outcomes. Further stratified analyses examining interpregnancy intervals and maternal age are warranted to disentangle these effects [33, 34]. Emergency cesarean delivery was highlighted by DL models as a potential risk marker, reflecting perinatal distress scenarios.

Interestingly, neither consanguinity nor gestational diabetes mellitus (GDM) emerged as statistically significant predictors of CP in this cohort. These findings merit careful, critical reflection rather than dismissal, as both variables have prior literature support, and both null results carry important interpretive uncertainty. For consanguinity, several established CP-associated recessive loci have been reported at higher frequencies in consanguineous Middle Eastern families, including variants in TUBA1A and related tubulinopathy genes [35], genes encoding subunits of the adaptor protein complex-4 (AP4E1, AP4M1, AP4B1, AP4S1) identified in consanguineous Palestinian-Jordanian and Pakistani families [36], and a broader catalogue of recessive CP-associated genes recently characterised across 188 primarily consanguineous Middle Eastern families, in whom putative causative recessive variants were identified in nearly three-quarters of cases [37]. The absence of a significant association in the present study most likely reflects a statistical power limitation: with a background consanguinity prevalence of approximately 54% in Qatar [38, 39], the contrast between cases and controls is inherently compressed, and a sample of *n* = 381 CP cases would have substantially reduced power to detect odds ratios of the magnitude typically reported for consanguinity in Middle Eastern CP populations (OR 2.5–4.6) [8, 40]. A post-hoc power calculation suggests that detecting an OR of 1.5 with 80% power at α = 0.05 against a 55% baseline prevalence would require approximately 600 cases and 1,000 controls, well above our current sample. We therefore do not interpret the null finding as evidence against a consanguinity effect; rather, it is most parsimoniously explained by insufficient power in the context of a high-prevalence exposure. Larger, registry-linked studies with detailed pedigree data and genetic validation are needed to resolve this. For GDM, the literature is less consistent than for consanguinity. Some cohort and registry-based studies report a modest-to-moderate elevation in CP risk: a large Norwegian registry study found GDM associated with a 1.8-fold excess risk of CP (RR = 1.8, 95% CI: 1.4–2.4) [41], and a two-sample Mendelian randomisation study using GWAS summary data estimated a causal OR of 1.74 (95% CI: 1.27–2.37, *p* < 0.001) for genetically predicted GDM on offspring CP risk [42]. In contrast, a population-based birth cohort of over 2.1 million live births in Ontario found no association between GDM and CP after adjustment for maternal sociodemographic and clinical factors (adjusted HR = 0.91, 95% CI: 0.77–1.06), whereas pregestational diabetes was significantly associated (adjusted HR = 1.84) [43] – directly illustrating how the GDM–CP signal can disappear once correlated maternal metabolic conditions are adequately controlled. The absence of significance in our dataset may therefore represent a genuine contextual finding, particularly given that Qatar carries one of the highest GDM burdens globally (pooled prevalence 20.7%, 95% CI: 15.2–26.7%) driven primarily by the high background prevalence of obesity and type 2 diabetes rather than pregnancy-specific metabolic dysfunction per se [44, 45]. In such a population, where metabolic comorbidities are highly prevalent and co-linearly distributed across both cases and controls, the marginal independent contribution of GDM to CP risk may be genuinely attenuated relative to populations where GDM occurs against a lower background metabolic burden. This interpretation is speculative but biologically plausible and is consistent with the known difficulty of disentangling GDM from obesity and advanced maternal age in regression models. We acknowledge that both explanations, insufficient power and genuine biological attenuation, remain viable, and explicitly flag this as a priority area for replication in adequately powered studies with detailed metabolic phenotyping.

Risk interaction analysis showed a cumulative effect. Infants with multiple exposures (Group 16) had a CP risk exceeding 93%, demonstrating the advantage of RF classifiers over traditional LR for capturing complex, nonlinear associations. GMFCS-based severity analyses found NICU admission, LBW, and GA consistent predictors of severe motor impairment. These findings confirm known perinatal risks and establish ML-driven methods as valuable tools for early CP risk stratification and intervention planning.

### Strengths and limitations

This study benefits from a well-characterized, retrospectively collected clinical dataset (381 CP cases, 660 controls) that, while modest in size for complex ML interaction modelling, represents one of the largest CP cohorts from the Middle East. The application of advanced ML models enhances the prediction of CP risk based on perinatal and clinical factors. Ensemble methods improved performance and interpretability, while cross-validation ensured model stability; however, the single-center, retrospective design limits generalizability. Recruitment from a rehabilitation setting may have skewed the sample toward more severe cases. The FFN model demonstrated strong discriminative performance (ROC-AUC = 0.9758, PR-AUC = 0.9719) with no evidence of overfitting, attributed to dropout regularization applied after each hidden layer; however, its interpretability is inherently more limited than the tree-based models. Among the three ML classifiers, SVM achieved the highest cross-validated ROC-AUC (0.978 ± 0.006) and F1-score, followed closely by RF and XGBoost (ROC-AUC 0.971 and 0.972, respectively), with overlapping confidence intervals suggesting that differences in performance were modest and may not be clinically meaningful. Additional constraints include the absence of genetic and socioeconomic data, a limited follow-up period, and reliance on non-CP pediatric controls. Mild CP may be underrepresented, and preterm births overrepresented, due to referral patterns. Future work should include community-based, prospective cohorts with broader variable integration to refine prediction and improve generalizability.

## Conclusion

This study demonstrates that a multidimensional ML framework integrating perinatal, clinical, and familial variables can predict CP risk with high accuracy in a Middle Eastern pediatric cohort. All four models (RF, XGBoost, SVM, and FFN) achieved ROC-AUC values of 0.98–0.99, with SHAP analysis confirming consistent cross-model importance of low birth weight, very preterm birth, NICU admission, and multigravidity. Cumulative interaction modelling across 16 exposure subgroups revealed a marked dose-response gradient: infants exposed to all four risk factors had a CP incidence of 93.15% (OR = 1382.67; *p* < 0.0001), demonstrating that these perinatal exposures act synergistically rather than additively. Severity analysis further confirmed NICU admission, low birth weight, and gestational age as independent predictors of higher GMFCS classification. Consanguinity and gestational diabetes mellitus did not reach statistical significance, most plausibly attributable to limited power against a high-prevalence exposure background in this population. While the retrospective, single-centre design limits generalizability, these findings support a paradigm shift from single-factor screening toward exposure-weighted, ML-driven risk stratification in neonatal surveillance. Future prospective, multi-center studies incorporating genetic and socioeconomic data are needed to validate and extend these findings.

## Supplementary Information

Below is the link to the electronic supplementary material.

Supplementary Material 1

Supplementary Material 2

## Data Availability

The data supporting the findings of this study are available from Sidra Medicine’s REDCap platform and the CERN database. Access to this data is restricted due to privacy and ethical considerations. Data can be made available from the corresponding author on reasonable request and approval from Sidra Medicine and Hammad Bin Khalifa University under the research protocol NPRP14S-0319-210075 (Sidra-IRB-1801339, QBRI-IRB-2024-6). The code used for this study, including implementations of ML models and NN, is available at [https://github.com/Foysalgebt12/CP].
