# Supplementary material for: Machine learning driven modeling of synergistic perinatal risk profiles in early onset pediatric cerebral palsy
Source: BMC Med Inform Decis Mak. 2026 Jul 11;26:437. doi: 10.1186/s12911-026-03710-y (PMC13715290; doi:10.1186/s12911-026-03710-y)
Supplement: Supplementary file 2 [file 12911_2026_3710_MOESM2_ESM.docx]

**Supplementary Content**

**Table S1.** Descriptive summary of the clinical and demographic characteristics of the study population, categorized into CP and Non-CP groups.

| **Variable** | **CP (n) = 381** | **Non-CP (n) = 660** |
| --- | --- | --- |
| **Gender** |  |  |
| Male | 255 (66.93%) | 387 (58.64%) |
| Female | 126 (33.07%) | 273 (41.36%) |
| **Age (Year)** |  |  |
| Mean (SD) | 9.44 (5.23) | 8.43 (5.34) |
| 95% CI | 8.92 - 9.97 | 7.37 - 8.19 |
| **Nationality** |  |  |
| Qatari | 123 (32.28%) | 258 (67.72 %) |
| Non-Qatari | 256 (38.79% ) | 404 (61.21%) |
| **GMFCS** |  |  |
| I | 82 (26.8%) |  |
| II | 52 (17.0%) |  |
| III | 28 (9.15%) |  |
| IV | 16 (5.23 %) |  |
| V | 128 (41.83%) |  |
| **Motor Types** |  |  |
| Ataxic | 6 (1.9%) |  |
| Dyskinetic | 1 (0.39%) |  |
| Dystonic | 5 (1.6 %) |  |
| Hypotonic | 14 (4.4%) |  |
| Mixed | 2 (0.6%) |  |
| Spastic | 289 (91.2%) |  |
| **Topography** |  |  |
| Diplegic | 87 (31.8%) |  |
| Hemiplegia | 79 (28.8%) |  |
| Quadriplegic | 98 (35.8%) |  |
| Triplegic | 10 (3.6%0 |  |
| **Gravidity** |  |  |
| Multigravida | 242 (63.52%) | 113 (17.12%) |
| Primigravida | 139 (36.48%) | 547 (82.88%) |
| **Number of Pregnancy (Mother)** |  |  |
| Mean (SD) | 2.19 (1.58) | 1.48 (1.32) |
| 95% CI | 2.03-2.35 | 1.0 (1.33-1.50) |
| **Parity** |  |  |
| Multiparous | 192 (50.39) | 113 (17.12%) |
| Primiparous | 189 (49.61) | 547 (82.88%) |
| **Number of Children** |  |  |
| Mean (SD) | 2.25 (1.59) | 1.42 (1.09) |
| 95% CI | 2.09 - 2.41 | 1.33 - 1.50 |
| **Mother Age During Birth (Year)** |  |  |
| Mean (SD) | 28.21 (3.08) | 28.63 (3.31) |
| 95% CI | 27.90 - 28.52 | 28.37 - 28.88 |
| **Maternal Health** |  |  |
| Gestational Diabetes | 337 (88.45%) | 600 (90.91%) |
| Other | 44 (11.55%) | 60 (9.09%) |
| **Gestation Status** |  |  |
| Full term | 48 (12.60%) | 573 (86.82%) |
| Premature | 333 (87.40%) | 87 (13.18%) |
| **Term, n(%)** | 341 | 340 |
| Early term | 15 (3.94%) | 8 (1.21%) |
| Full term | 48 (12.60%) | 575 (87.12%) |
| Mild preterm | 32 (8.40%) | 49 (7.42%) |
| Very preterm | 286 (75.07%) | 28 (4.24%) |
| **Gestation Age (weeks)** | 341 | 340 |
| Mean (SD) | 29.66, SD: 5.39 | 39.08, SD: 3.06 |
| Min, Max | 29.12 - 30.20 | 38.85 - 39.32 |
| **Delivery Mode** |  |  |
| Elective cesarean section | 76 (19.95%) | 157 (23.79%) |
| Emergency cesarean section | 190 (49.87%) | 67 (10.15%) |
| Vaginal | 115 (30.18%) | 436 (66.06%) |
| **Birth Weight (Gram)** |  |  |
| Mean (SD) | 2019.40, (638.37) | 3282.13 (752.83) |
| 95% CI | 1955.09 - 2083.70 | 3224.59 - 3339.67 |
| **Birth Weight Category (BWC)** |  |  |
| Low BW | 344 (90.29%) | 86 (13.03%) |
| Normal BW | 34 (8.92%) | 479 (72.58%) |
| High BW | 3 (0.79%) | 95 (14.39%) |
| **Admitted to NICU** | 172 | 298 |
| No | 52 (13.65%) | 423 (64.09%) |
| Yes | 329 (86.35%) | 237 (35.91%) |
| **Past Surgeries of Mother** | 142 | 324 |
| No | 131 (34.38%) | 360 (54.55%) |
| Yes | 250 (65.62%) | 300 (45.45%) |
| **Gtube of Children** |  |  |
| No | 329 (86.35%) | 60 (10.3%) |
| Yes | 52 (13.65%) | 56 (8.48%) |
| **Consanguine marriage** |  |  |
| No | 216 (56.69%) | 604 (91.52%) |
| Yes | 165 (43.31%) | 322 (48.79%) |
| **Physical therapy** |  |  |
| No | 87 (22.83%) | 323 (48.94%) |
| Yes | 294 (77.17%) | 337 (51.06%) |

**Footnote: “Number of Children” refers to total children born to mother.**

**Table S2.** The p-values indicate the level of statistical significance, with values less than < 0.05 considered significant. Results are formatted to show original p-values with thresholds: p < 0.05, p < 0.01, and p < 0.001, where applicable.

| **Variable** | **P-value** | **Formatted P-value** |
| --- | --- | --- |
| Gender | 0.009743418 | <0.01 |
| GMFCS | 1 | 1 |
| Age | 1.24249E-06 | < 0.05 |
| Motor Types | 1 | 1 |
| Topography | 1 | 1 |
| Gravidity | 8.38838E-52 | <0.001 |
| Number of Pregnancy | 2.28686E-14 | <0.001 |
| Parity | 1.44787E-29 | <0.001 |
| Number of Children | 1.3606E-22 | <0.001 |
| Mother Age | 0.047177225 | < 0.05 |
| Mother Health | 0.243414774 | 0.243 |
| Gestation Status | 1.4028E-121 | <0.001 |
| Term | 3.7619E-137 | <0.001 |
| Gestation Age of baby (weeks) | 9.0866E-185 | <0.001 |
| Delivery Mode | 2.41575E-47 | <0.001 |
| Birth Weight | 1.1161E-125 | <0.001 |
| BWC | 5.8014E-130 | <0.001 |
| Admit to NICU | 2.23075E-55 | <0.001 |
| Past Surgeries | 5.20118E-10 | <0.001 |
| G tube | 0.011528378 | < 0.05 |
| Consanguine Marriage | 0.100440369 | 0.1 |
| Physcial Therapy | 1.75292E-16 | < 0.001 |

**Table S3.** Univariate and multivariate logistic regression analysis of clinical and perinatal variables associated with CP. The table reports odds ratios (OR), 95% confidence intervals (CI), and p-values for each variable. Univariate models evaluate each variable independently, while multivariate models adjust for all other covariates in the model. Significant associations (p < 0.05) are highlighted.

| **Model** | **Variable** | **Odds Ratio [95% CI]** | **p-value** |
| --- | --- | --- | --- |
| Univariate | Geastation_Status | 0.02 [0.02–0.03] | 1.13e-87 |
| Univariate | Admit_to_NICU | 0.09 [0.06–0.12] | 3.32e-46 |
| Univariate | Term | 0.13 [0.11–0.16] | 1.65e-82 |
| Univariate | Physcial_Therapy | 0.31 [0.23–0.41] | 4.74e-16 |
| Univariate | Past_Surgeries | 0.44 [0.34–0.57] | 4.97e-10 |
| Univariate | G_tube | 0.59 [0.39–0.88] | 0.00907 |
| Univariate | Number_of_Childerens | 0.62 [0.56–0.69] | 1.39e-18 |
| Univariate | Gender | 0.70 [0.54–0.91] | 0.00815 |
| Univariate | Number_of_Prgenancy | 0.70 [0.64–0.77] | 1.94e-12 |
| Univariate | Mother_Health | 0.77 [0.51–1.16] | 0.204 |
| Univariate | Birth_Weight | 1.00 [1.00–1.00] | 4.45e-61 |
| Univariate | Mother_Age | 1.04 [1.00–1.08] | 0.0482 |
| Univariate | Consanguine_Marriage | 1.25 [0.97–1.61] | 0.088 |
| Univariate | Gestation_Age_of_baby_(weeks) | 1.44 [1.38–1.50] | 8.3e-75 |
| Univariate | Delivery_Mode | 1.62 [1.39–1.89] | 1.37e-09 |
| Univariate | BWC | 3.45 [2.78–4.29] | 5.76e-29 |
| Univariate | Parity | 4.92 [3.70–6.54] | 6.99e-28 |
| Univariate | Gravidity | 8.43 [6.30–11.27] | 8.06e-47 |
| Multivariate | Physcial_Therapy | 0.45 [0.26–0.76] | 0.00315 |
| Multivariate | Geastation_Status | 0.48 [0.17–1.30] | 0.146 |
| Multivariate | Admit_to_NICU | 0.51 [0.29–0.89] | 0.0167 |
| Multivariate | Gender | 0.58 [0.33–1.02] | 0.0591 |
| Multivariate | Consanguine_Marriage | 0.75 [0.45–1.26] | 0.277 |
| Multivariate | Past_Surgeries | 0.75 [0.42–1.35] | 0.341 |
| Multivariate | Number_of_Childerens | 0.92 [0.59–1.44] | 0.709 |
| Multivariate | Birth_Weight | 1.00 [1.00–1.00] | 1.48e-05 |
| Multivariate | Delivery_Mode | 1.02 [0.71–1.46] | 0.933 |
| Multivariate | Mother_Age | 1.06 [0.97–1.15] | 0.184 |
| Multivariate | Term | 1.10 [0.59–2.05] | 0.772 |
| Multivariate | Number_of_Prgenancy | 1.31 [0.91–1.88] | 0.15 |
| Multivariate | Gestation_Age_of_baby_(weeks) | 1.32 [1.14–1.53] | 0.000245 |
| Multivariate | G_tube | 1.89 [0.88–4.03] | 0.101 |
| Multivariate | BWC | 2.44 [1.60–3.73] | 3.46e-05 |
| Multivariate | Mother_Health | 4.97 [2.27–10.87] | 6.04e-05 |

**Table S4.** The variance inflation factor (VIF) values for each feature used in the LR model to predict outcomes related to CP versus non-CP.

| **Features** | **VIF** |
| --- | --- |
| Gender | 2.779391 |
| Gravidity | 16.214021 |
| Number_of_Prgenancy | 9.649909 |
| Parity | 25.285581 |
| Number_of_Childerens | 12.614201 |
| Mother_Age | 74.757726 |
| Mother_Health | 1.179507 |
| Geastation_Status | 7.755684 |
| Term | 17.925191 |
| Gestation_Age_of_baby_(weeks) | 96.076112 |
| Delivery_Mode | 4.832631 |
| Birth_Weight | 23.007237 |
| BWC | 6.534908 |
| Admit_to_NICU | 3.091664 |
| Past_Surgeries | 2.893786 |
| G_tube | 1.219581 |
| Consanguine_Marriage | 1.943788 |
| Physcial_Therapy | 2.820742 |

**Table S5.** Performance metrics for the different ML models. Metrics include ROC-AUC, PR-AUC, F1-score, and recall for Random Forest, XGBoost, and SVM. Bold values highlight the best-performing model for each metric.

| Model | ROC_AUC | PR_AUC | F1_Score | Recall | Precision | TN | FP | FN | TP |
| --- | --- | --- | --- | --- | --- | --- | --- | --- | --- |
| RandomForest | 0.983018 | 0.97283 | 0.910053 | 0.895833 | 0.924731 | 158 | 7 | 10 | 86 |
| XGBoost | 0.980997 | 0.966495 | 0.910053 | 0.895833 | 0.924731 | 158 | 7 | 10 | 86 |
| SVM | 0.990025 | 0.983107 | 0.927835 | 0.9375 | 0.918367 | 157 | 8 | 6 | 90 |

**Table S6.** This table displays the relative importance of features with the relative importance score for predicting CP across different ML models.

| **Model** | **Features** | **Relative Importance** | **Indicate** |
| --- | --- | --- | --- |
| Random Forest | BWC_Low | 0.173633173 | CP |
| Random Forest | Term_Very preterm | 0.14788005 | CP |
| Random Forest | Geastation Status_Full term | 0.09610935 | Non-CP |
| Random Forest | Geastation Status_Premature | 0.082661431 | CP |
| Random Forest | Term_Full term | 0.072044584 | Non-CP |
| Random Forest | BWC_Normal | 0.057449867 | Non-CP |
| Random Forest | Gravidity_Multigravida | 0.054226804 | CP |
| Random Forest | Gravidity_Primigravida | 0.040453176 | Non-CP |
| Random Forest | Admit to NICU_Yes | 0.029769102 | CP |
| Random Forest | Delivery Mode_Emergency cesarean section | 0.02940601 | CP |
| XGB | BWC_Low | 0.605840921 | CP |
| XGB | Term_Very preterm | 0.09519843 | CP |
| XGB | Term_Early term | 0.094670817 | CP |
| XGB | Gravidity_Multigravida | 0.070938148 | CP |
| XGB | Parity_Multiparous | 0.022851232 | CP |
| XGB | Term_Full term | 0.021416746 | Non-CP |
| XGB | G tube_No | 0.0115325 | Non-CP |
| XGB | Admit to NICU_Yes | 0.010468642 | CP |
| XGB | Geastation Status_Full term | 0.010410436 | Non-CP |
| XGB | Gender_Female | 0.008251164 | Non-CP |
| SVM | Gravidity_Primigravida | 0.97479104 | Non-CP |
| SVM | Gravidity_Multigravida | 0.940709661 | CP |
| SVM | Term_Very preterm | 0.639810088 | CP |
| SVM | Parity_Multiparous | 0.602610004 | CP |
| SVM | Parity_Primiparous | 0.568528624 | Non-CP |
| SVM | Term_Mild preterm | 0.414064552 | CP |
| SVM | BWC_Low | 0.405367279 | CP |
| SVM | Term_Full term | 0.317964477 | Non-CP |
| SVM | Admit to NICU_Yes | 0.275802607 | CP |
| SVM | BWC_High | 0.163646052 | Non-CP |

**Table S7.** Group Categorization Based on Risk Factors. Each neonate was categorized into one of 16 distinct groups based on the presence or absence of fourkey perinatal risk factors: preterm birth (<37 weeks gestation), low birth weight (<2500g), and NICU admission, Multipregnancy (>1).

| **Group** | **OR** | **CI_lower** | **CI_upper** | **p_value** | **Group Description** | **combined effect (%)** |
| --- | --- | --- | --- | --- | --- | --- |
| G1 | 1 |  |  |  | Full-term, Normal BW, No NICU | 0.97 |
| G2 | 15.14184 | 3.78293 | 60.60786 | 9.63E-05 | Multipreg | 12.96 |
| G3 | 2.13287 | 0.42522 | 10.69827 | 3.92E-01 | NICU | 2.05 |
| G4 | 33.88889 | 8.99706 | 127.648 | 9.60E-09 | NICU + Multipreg | 25 |
| G5 | 5.80952 | 0.93838 | 35.96684 | 9.10E-02 | Low BW | 5.41 |
| G6 | 338.8889 | 60.69372 | 1892.217 | 2.87E-14 | Low BW + Multipreg | 76.92 |
| G7 | 83.18182 | 19.73816 | 350.5501 | 3.10E-10 | Low BW + NICU | 45 |
| G8 | 321.9444 | 74.66526 | 1388.172 | 6.28E-23 | Low BW + NICU + Multipreg | 76 |
| G9 | 74.55556 | 18.79694 | 295.7147 | 2.02E-11 | Preterm | 42.31 |
| G10 | 101.6667 | 16.92778 | 610.6005 | 5.83E-06 | Preterm + Multipreg | 50 |
| G11 | 258.7879 | 68.17005 | 982.4133 | 4.72E-29 | Preterm + NICU | 71.79 |
| G12 | 101.6667 | 18.90654 | 546.695 | 5.16E-07 | Preterm + NICU + Multipreg | 50 |
| G13 | 124.2593 | 29.48541 | 523.6612 | 5.65E-13 | Preterm + Low BW | 55 |
| G14 | 81.33333 | 14.29928 | 462.6184 | 1.03E-05 | Preterm + Low BW + Multipreg | 44.44 |
| G15 | 666.4815 | 192.7521 | 2304.502 | 1.56E-83 | Preterm + Low BW + NICU | 86.76 |
| G16 | 1382.667 | 374.5808 | 5103.75 | 3.68E-99 | Preterm + Low BW + NICU + Multipreg | 93.15 |

**Table S8.** Logistic Regression Results for CP Prediction Using Key Perinatal Risk Factors This table presents the coefficients, standard errors, z-scores, p-values, and 95% confidence intervals from the final multivariable logistic regression model.

| **Variable** | **Coef.** | **Std.Err.** | **z** | **P>\|z\|** | **[0.025** | **0.975]** |
| --- | --- | --- | --- | --- | --- | --- |
| GestationAgeofbaby(weeks) | -0.13237 | 0.024194 | -5.47115 | 4.47E-08 | -0.17979 | -0.08495 |
| Birth_Weight_kg | -0.57031 | 0.122946 | -4.63869 | 3.51E-06 | -0.81128 | -0.32934 |
| Admit_NICU_Binary | 2.028273 | 0.188664 | 10.7507 | 5.88E-27 | 1.658498 | 2.398048 |
| Number of _Pregnancy | -0.04862 | 0.047189 | -1.0303 | 0.302869 | -0.14111 | 0.04387 |

**Figure S1. Pie chart shows the proportion of individuals diagnosed with CP compared to those without Non-CP. Pie represents the percentage and actual count of cases in the Non-CP category.**

**Figure S2.** Performance metrics of the logistic regression (LR) model for cerebral palsy classification. The model demonstrated strong discriminative capacity, achieving an AUC-ROC of 0.9700. Additional evaluation metrics included a precision of 90.3%, recall of 81.3%, and an F1-score of 85.5%, highlighting its balanced predictive power and reliability in identifying CP cases.

**Figure S3.** Represent the features selection process. (A). Displays mean differences in scaled feature values between CP and non-CP groups. (B) Violin plots explaining the distribution of the top features by outcome group. (C) Boxplots provide a comparative view of value spread for each top feature. (D) Heatmap depicts Pearson correlations among the top predictive features, with stronger red or blue hues indicating higher positive or negative correlations.

**Figure S4.** SHAP-Based Feature Importance Interpretation Across ML Models.
SHAP summary plots (left column) and beeswarm plots (right column) illustrate the relative influence of input features on CP prediction across three machine learning classifiers: (A–B) Random Forest (RF), (C–D) XGBoost, and (E–F) SVM. Summary plots rank features by mean absolute SHAP value, reflecting average contribution to model output. Beeswarm plots display individual-level SHAP impacts, with color indicating feature value magnitude (pink = high, blue = low).

**Figure S5.** Performance evaluation of the DNN model. (A) Bar plot summarizing key performance metrics, including Recall, F1 Score, and PR-AUC, indicating high sensitivity and balanced precision-recall tradeoff. (B) Precision-Recall curve demonstrating the model’s ability to distinguish between CP and non-CP.

**Figure S6**. Evaluation of model performance using precision-recall and classification metrics. (A) Precision-Recall curve showing high PR-AUC (0.9632). (B–D) Bar plots of recall, precision, and F1 score, indicating strong and balanced classification performance on -adjusted data.

**Figure S7**. Multivariable Modeling and Diagnostic Evaluation of CP Severity Predictors (A) Ordinal Logistic Regression for GMFCS I–V Severity Scale: Forest plot depicting adjusted odds ratios and 95% confidence intervals for key perinatal and demographic variables. (B) Binary Logistic Regression Stratified by Severity: Severity defined as GMFCS IV–V versus I–III. (C) ROC Curve for Logistic Regression Model. (D) Multicollinearity Assessment via VIF, for all key predictors show values below 2.0, indicating minimal multicollinearity among included features.
